# Bismuth Bicycles

**DOI:** 10.1002/psc.70071

**Published:** 2026-02-03

**Authors:** Saan Voss, Amin Sagar, Arnaud Tiberghien, Richard J. L. Hughes, Liuhong Chen, Inmaculada Rioja, Mark Frigerio, Michael J. Skynner, David R. Spring

**Affiliations:** ^1^ Yusuf Hamied Department of Chemistry University of Cambridge Cambridge UK; ^2^ Bicycle Therapeutics Portway Building, Granta Park Cambridge UK

## Abstract

Bicyclic peptides are emerging as next generation therapeutics by combining the affinity and specificity of antibodies with the synthetic convenience of small molecules. Phage‐encoded libraries of bicyclic peptides enable the discovery of high‐affinity molecules against virtually any protein target. The generation of bicyclic peptides that advanced into clinical development involves the reaction of three cysteines in a peptide to a C_3_‐symmetric alkylating agent. In phage display, this chemical modification transforms a pool of conformationally flexible peptides into a library of structurally unique protein mimetics that are able to bind traditionally challenging protein surfaces like those with limited structural definition. In recent years, a new class of bicyclic peptides has emerged using a single atom—bismuth—in place of C_3_‐symmetric organic scaffolds, thus expanding into an unexplored chemical space at the intersection of inorganic chemistry and biology. This mini‐review aims to reflect on the discovery, evolution and potential future applications of bismuth bicycle molecules.

## Graphical abstract

Discovery, evolution and potential future applications of bismuth bicycle molecules—a novel class of therapeutics with unique properties.

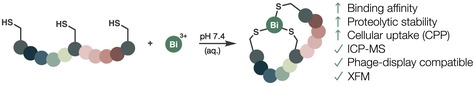

## 1 Introduction

**FIGURE 1:**
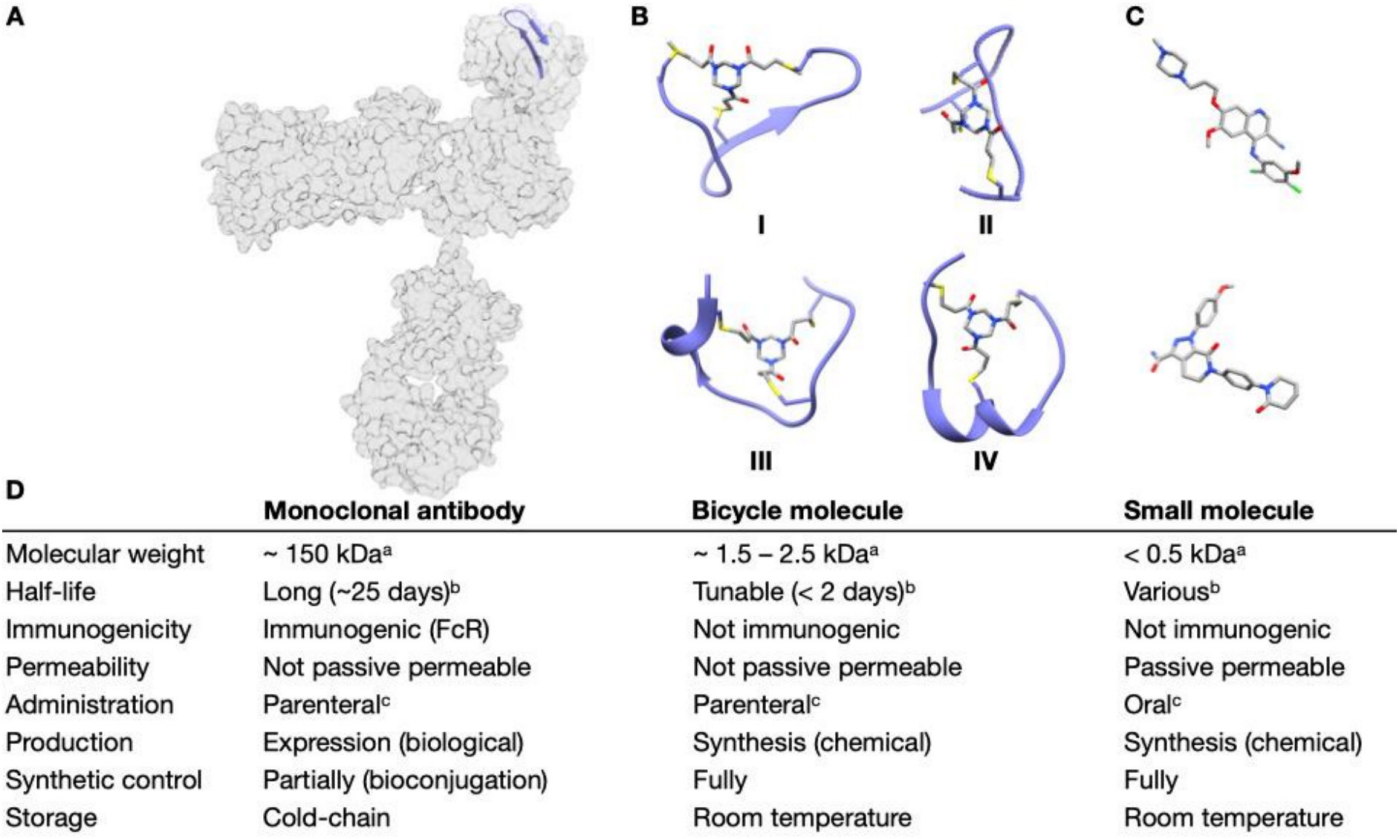
Comparison of different therapeutics modalities. (A) Surface map of an IgG antibody shown in light grey (accession code: 1IGT). Cartoon structure of a β‐hairpin within the light chain comprising 15 amino acids is highlighted in purple to allow for a relative comparison of the size of an antibody to a Bicycle molecule. Each of the Bicycle molecules I‐IV comprise no more than 15 amino acids (excluding the scaffold). (B) Cartoon structures of four Bicycle molecules cyclised with TATA (1,3,5‐triacryloylhexahydro‐1,3,5‐triazine). (I) Bicycle molecule targeting CD137 (accession code: 6Y8K), (II) Bicycle molecule targeting E. coli PBP3 (accession code: 8RTZ), (III) Bicycle molecule targeting ACE2 (accession code: 8BN1), (IV) Bicycle molecule targeting EphA2 (accession code: 6RW2). Ribbon representation of amino acid backbone shown in purple, C_3_‐symmetric organic scaffold (TATA) shown in grey, heteroatoms highlighted in red (oxygen), blue (nitrogen) and yellow (sulfur). (C) 3D structures of Bosutinib (top) and Apixaban (bottom). Heteroatoms highlighted as in (B) with the addition of green (chloride). (D) Table comparing selected properties of antibodies, Bicycle molecules and small molecules.^a,b^ representative values [1, 2]—certain examples may deviate in their values^c^ Main route of administration. NB structures not drawn to scale.

Bicyclic peptides are emerging as next‐generation therapeutics, occupying a chemical space between antibodies and small molecules (Figure 1) [3, 4, 5, 6, 7]. Like antibodies, bicyclic peptides are known for their extraordinary affinity and specificity [3]. Yet akin to small molecules, bicyclic peptides are accessible through chemical synthesis allowing for a compound's alteration on an atomic level—to control pharmacokinetic parameters such as tissue penetration and clearance [8, 9, 10, 11]. Unlike antibodies, bicyclic peptides have shown no signs of immunogenicity to date, which avoids potential adverse effects associated with Fc‐related pharmacological effects (Figure 1).

**FIGURE 2:**
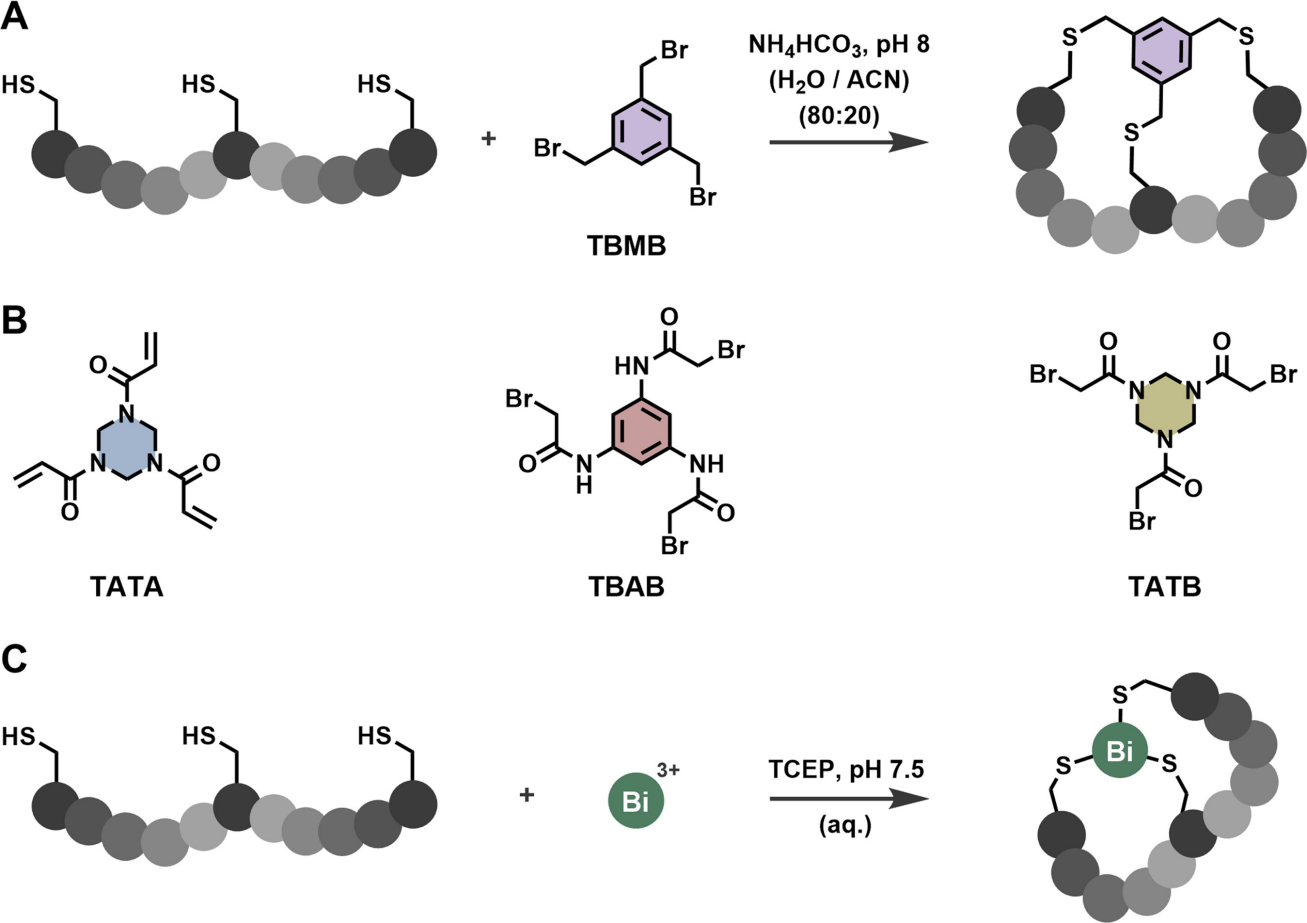
Schematic illustration comparing the synthetic scheme of Bicycle molecules generated with C_3_‐symmetric organic scaffolds and Bi^3+^. (A) Schematic reaction scheme for the synthesis of Bicycle molecules using TBMB. (B) Alternative C_3_‐symmetric organic scaffolds for cysteine alkylation: 1,3,5‐triacryloylhexahydro‐1,3,5‐triazine (TATA), N,N',N"‐benzene‐1,3,5‐triyltris(2‐bromoacetamide) (TBAB), 1,1',1"‐(1,3,5‐triazinane‐1,3,5‐triyl)tris(2‐bromoethan‐1‐one) (TATB). (C) Schematic reaction scheme for the synthesis of Bicycle molecules using Bi^3+^. Suitable salts for the synthesis of bismuth Bicycle molecules include BiBr_3_ (soluble in organic solvents including dimethyl sulfoxide or acetonitrile) and bismuth tripotassium dicitrate (soluble in water).

A variety of double‐looped peptidomimetics could be considered bicyclic peptides [7, 12, 13, 14, 15], but the topology which arguably defined the term in its recent incarnation is characterised by a linear peptide that is anchored at three joints to a central scaffold, forming a bicyclic product (Figures 1 and 2) [6, 12]. A prominent example involves the reaction of three cysteines in a native peptide to alkylating agents such as 1,3,5‐tris (bromomethyl)benzene (TBMB) (Figure 2A) [16, 17]. This chemical modification can improve both binding affinity and proteolytic stability in comparison to its linear congener [16, 18, 19].

Winter and co‐workers, at the MRC Laboratory for Molecular Biology (Cambridge, UK), pioneered phage‐encoded combinatorial chemical libraries of Bicycle molecules by developing engineered bacteriophages, which displayed semi‐randomised peptide sequences on their surface [16]. These N‐terminal extensions of the bacteriophage's pIII protein comprise three cysteines separated by multiple randomised residues. The subsequent modification of the three thiol groups with TBMB restricts the conformational flexibility and transforms short random‐coiled peptides into a library of structurally unique protein mimetics. Affinity based selections of these combinatorial libraries with a diversity of ~10^14^ against a given biological target enables the discovery of peptidomimetics with extraordinary affinity and specificity [16].

Bicycle Therapeutics, a pharmaceutical company founded on the work from Winter and Heinis [16], has been showcasing the power of this technology for 15 years. Within its late‐stage portfolio, Bicycle Therapeutics has a number of next‐generation therapeutics including a Bicycle Drug Conjugate (BDC) targeting Nectin‐4 (zelenectide pevedotin), in phase II/III clinical trials [10, 11, 20, 21, 22, 23, 24, 25]. These achievements were made possible through the continuous evolution of their phage‐display platform, which has introduced increased structural diversity through expansion of library formats and exploration of a range of C_3_‐symmetric organic scaffolds, including TATA, TBAB or TATB (Figure 2B) [26, 27, 28, 29, 30, 31, 32].

In recent years, a new evolution of scaffolds emerged using a single atom—bismuth—in place of C_3_‐symmetric organic scaffolds like TBMB [33]. This unconventional concept, thus expands into an unexplored chemical space of metal‐constrained Bicycle molecules which may hold unique opportunities for research and development. This mini‐review aims to reflect on the discovery, evolution and potential future applications of bismuth Bicycle molecules.

## 2 Bioinorganic Chemistry of Bismuth

Bismuth, the 83^rd^ element of the periodic table, was long believed to be the heaviest stable atom before its naturally occurring isotope (bismuth‐209) was shown to undergo alpha decay—with a half‐life (1.9 ± 0.2 × 10^19^ years) that exceeds the age of our universe by ~ 9 orders of magnitude [34, 35]. The predominant oxidation states are III and V, while Bi(III) is the most common and stable form [36]. The coordination number of Bi(III) complexes can vary from 3 to 10 resulting in a range of geometries [36, 37]. Stable complexes are known with organic ligands comprising carboxylates, amines and most favourably thiols [36, 38, 39, 40].

Bismuth's thiophilic nature makes it an attractive metal for selective modifications of cysteines [39]. Informative studies examined interactions between Bi^3+^ and glutathione (GSH), a naturally occurring tripeptide that possesses two carboxylic acids, a primary amine and a thiol [41]. Despite the presence of these alternate metal binding moieties, ^13^C, ^1^H NMR measurements showed exclusive binding of the thiolate to bismuth with a stoichiometry of 3:1 (Bi(GSH)_3_) from pH 2 to pH 10. The kinetics of the Bi‐S bond showed pH dependency, with slow exchange rates at pH 4 (3 s^−1^) and faster exchange rates at physiological pH 7.4 (1500 s^−1^). Considering a reported stability constant of log *K* ~ 30, this suggests that Bi (GSH)_3_ complexes are, at physiological pH, thermodynamically stable while kinetically labile [41].

A subsequent study examined Bi^3+^ binding to an N‐terminal domain of a cysteine‐rich protein [42]. The extracted decapeptide **1** (MPGCPCPGCG‐NH_2_) comprised three cysteines separated by either one or two residues. NMR measurements indicated exclusive binding of the three thiolates to bismuth. The resulting tridentate bismuth complex was stable from pH 2 to pH 10 in agreement with earlier work on Bi (GSH)_3_ and even in the presence of a competing nona‐peptide containing two vicinal cysteines (Ac‐ACCHDHKKH‐NH_2_). This increased stability is likely due to the chelate effect, which describes the greater stability of metal complexes with higher denticity (number of donor groups in a given ligand that bind to a metal centre) [43].

## 3 Bismuth Bicycle Molecules

**FIGURE 3:**
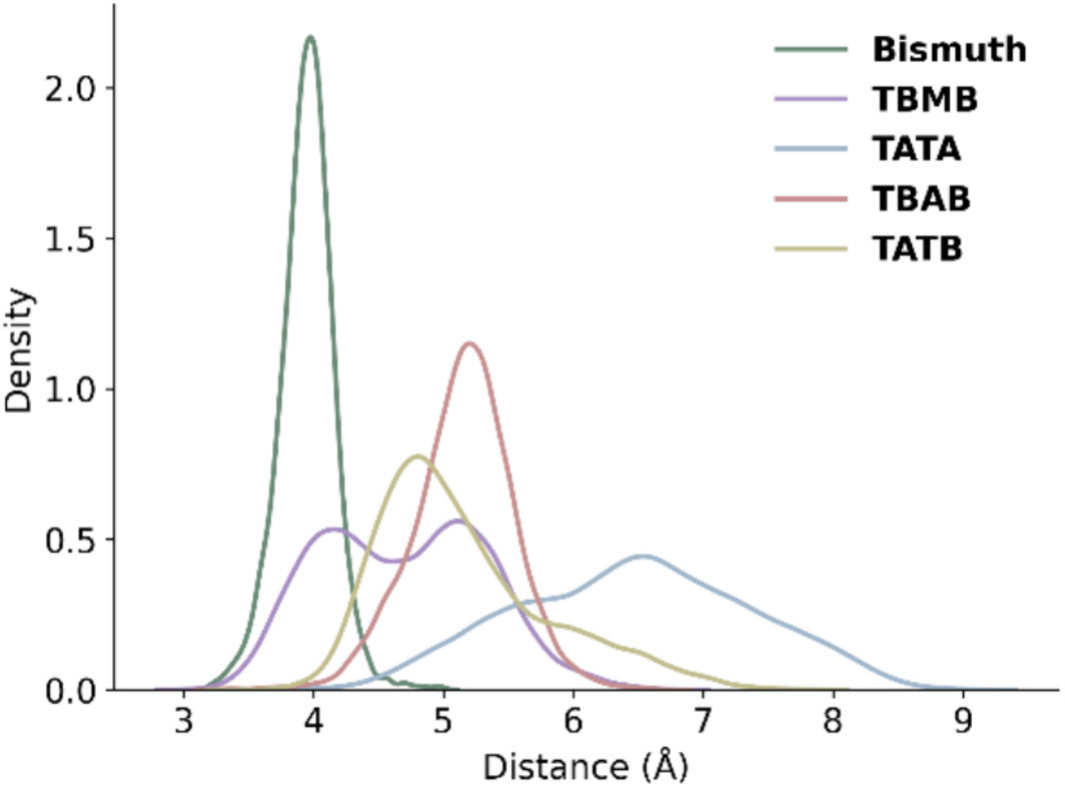
Distributions of the distances between the Cα of N‐acetyl cysteine methyl ester and the centre of selected scaffolds sampled by the simulations.

Encouraged by these and other examples [39, 44, 45, 46, 47, 48], Voss et al. set out to examine bismuth(III) as an alternative linker to generate a new class of Bicycle molecules (Figure 5) [33]. The reaction scheme proceeds in analogous fashion to those of conventional scaffolds like TBMB, which link three cysteines in a peptide (Figure 2). However, with an atomic radius of 1.5 Å [49], bismuth(III) represents the smallest and most constraint scaffold explored to generate Bicycle molecules (Figure 3 and Table 1).

**TABLE 1:** Mean distances between the Cα of N‐acetyl cysteine methyl ester and the centre of selected scaffolds.

Scaffold	Mean distance (Å)^a^ [standard deviation]	Interquartile range^b^
Bismuth	3.9 [+/− 0.2]	0.2
TBMB	4.7 [+/− 0.6]	1.0
TATA	6.5 [+/− 0.9]	1.3
TBAB	5.1 [+/− 0.4]	0.5
TATB	5.2 [+/− 0.7]	0.8

^a^ Average distance between the Cα of N‐acetyl cysteine methyl ester and the centre of a scaffold across the landscape of simulated conformations.

^b^ Difference between the 75th and 25th percentiles of the data.

In fact, for this work we ran computational simulations of Bi^3+^, TBMB, TATA, TBAB and TATB bound to three cysteines to compare the size and flexibility of the resulting conjugates. This allowed us to measure the distance between the C‐alpha atom (of each of the three cysteines) and the centre of each scaffold across the simulated landscape of conformations. Our data suggests that bismuth(III) forms the most constraint complexes with a mean distance of 3.9 Å and the narrowest distribution. At the other end of the spectrum is TATA, with a mean distance of 6.5 Å. The values for TBMB, TBAB, and TATB lie in between bismuth and TATA with 4.7, 5.1 and 5.2 Å, respectively (Figure 3 and Table 1).

A series of peptides following the general formula (CX_n_CX_m_C) with X_n_ and X_m_ being 3–8 amino acids was exposed to bismuth(III) at physiological pH and in presence of the reducing agent tris(2‐carboxyethyl)phosphine (TCEP). Liquid chromatography‐high resolution mass spectrometry data indicated in all cases the formation of a single product consistent with the mass of a bismuth Bicycle molecule. Importantly, the presence of sidechain moieties which might compete for bismuth binding, such as contained in aspartate, serine or histidine, also formed a single product. NMR measurements ([^13^C,^1^H]‐HSQC) of an isolated bismuth Bicycle molecule (**2**) confirmed the presence of a single species and showed exclusive binding of the three cysteines (Table 2) [33].

**TABLE 2:** Selected sequences of bismuth binding peptides

Cpd.	Sequence	Ref.
**1**	H‐MPGCPCPGCG‐NH_2_	42
**2**	Ac‐CKRKGCGKRKC‐NH_2_	33
**3**	RhB‐CKRKGCGKRKC‐NH_2_ ^a^	50
**4**	LACKRKGCAPYDCPG^b^	51
**5**	H‐AUPSDYUKRKGUG‐NH_2_	52
**6**	Ac‐AUPHPQUEAAAU‐NH_2_	52

^a^ RhB, Rhodamine B piperazine succinic acid.

^b^ Compound **4** has a macrocyclic backbone (head‐to‐tail cyclised through amide bond formation between its N‐terminal α‐amine and C‐terminal carboxylic acid).

Competition studies in the presence of GSH, at physiological pH, showed that bismuth Bicycle molecules tolerate up to 100 equivalents, although continued exposure (to 100 eq. GSH) over the course of two days resulted in partial dissociation [50], which can be attributed to the chelate effect and the above‐mentioned kinetic lability. The hexadentate chelator EDTA on the other hand, can displace the tridentate peptide ligand more rapidly. But a 2‐fold excess of EDTA did not promote complete bismuth dissociation from its peptide ligand [33]. The stability of bismuth peptide complexes can be further improved by converting the acyclic peptide chain into a monocyclic peptide precursor (by head‐to‐tail cyclisation) prior to bismuth binding which protects **4** largely against a 5‐fold excess of EDTA [51]. Another study suggests the use of selenocysteine in place of the three cysteines. The resulting selenocysteine bismuth Bicycle molecule **5** appears to tolerate 100 eq. EDTA (a hexadentate chelator) while **6** is reported to withstand 25 eq. of an octadentate analogue (DTPA) following 1h incubation at room temperature [52]. An explanation for these observations may be found in the principles of Hard and Soft Acids and Bases (HSAB), which describe the favourable interaction of soft Lewis acids like Bi(III) [53] with soft Lewis bases such as thiolates (as opposed to hard Lewis bases like carboxylates or alkylamines) [54, 55]. The HSAB principles also predict the greater strength of the bismuth‐selenium bond in which selenolates act as even softer Lewis bases, that trump the greater denticity of EDTA or DTPA [52, 54, 55].

**FIGURE 4:**
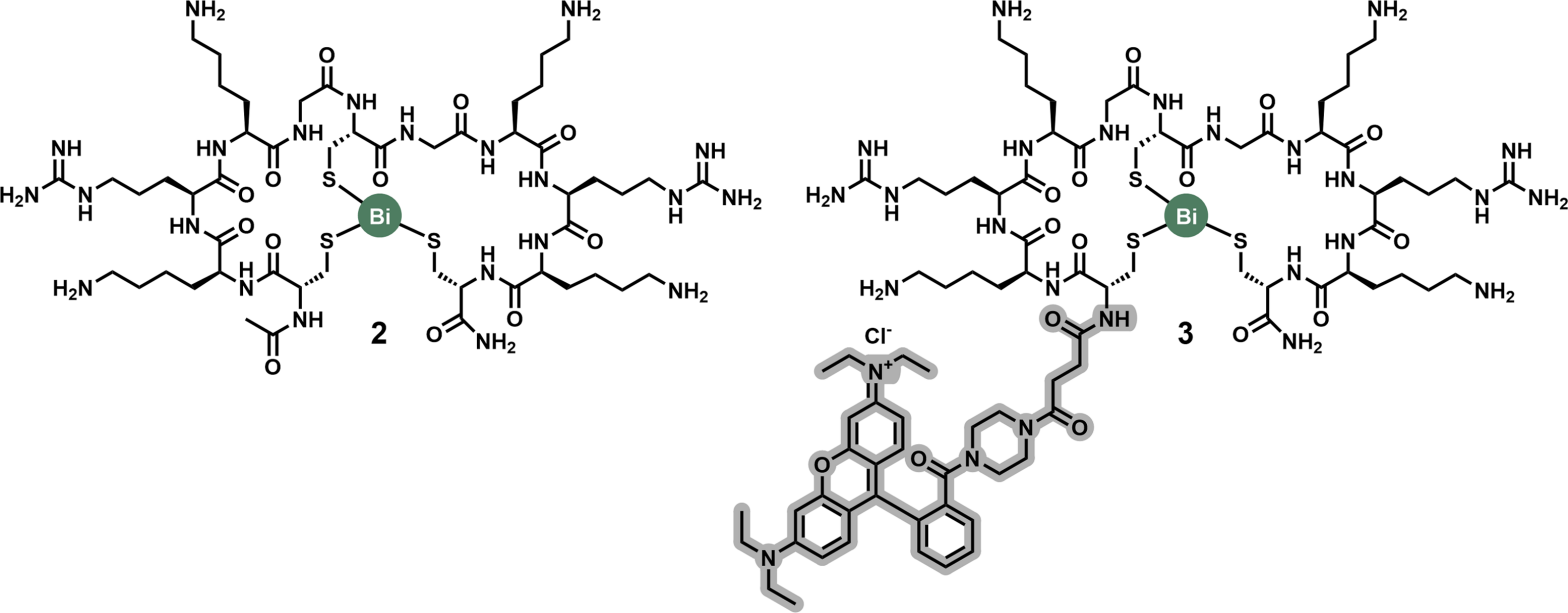
Chemical structures of selected bismuth Bicycle molecules **2** and its fluorescently labelled analogue **3**. Compound **2** and **3** are sequence analogues which differ in their N‐terminal extension. Compound **3** carries a rhodamine B derivative highlighted in grey.

**FIGURE 5:**
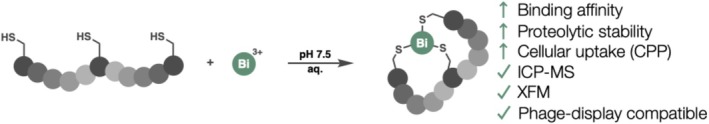
Schematic illustration showing the conversion of linear peptides comprising three cysteines to its corresponding bismuth Bicycle molecule under biocompatible conditions. Linear peptides comprising three cysteines react in the presence of the reducing agent TCEP with Bi^3+^ salts under biocompatible conditions instantaneously to the corresponding bismuth Bicycle molecule. Bismuth bicyclisation improves binding affinity, increases proteolytic stability and enhances cellular uptake of cell‐penetrating peptides (CPPs). Bismuth Bicycle molecules can be quantified from biological matrices directly using inductively coupled plasma‐mass spectrometry (ICP‐MS), bypassing the need for an additional reporter system such as fluorescent labels. Another technique that leverages the bismuth core directly as a reported system is X‐ray fluorescence microscopy (XFM), which not only enables quantification from biological matrices but allows localisation of bismuth Bicycle molecules at subcellular level. Bi^3+^ salts are compatible with phage display, thus enabling access to a chemical space at the intersection of inorganic chemistry and biology.

The introduction of structural constraint through peptide cyclisation can favour a conformation that complements the target binding site [13, 56]. This preorganisation can result in a reduced entropic penalty upon binding, which translates into improved binding affinity [16, 19]. To examine whether bismuth binding has a comparable effect on a peptide's biological activity, a screening campaign against proteases from Zika [57] and West Nile viruses [58] was conducted. A series of 15 rationally designed protease inhibitors bearing either one or two substrate recognition motifs were screened in situ against both targets. These efforts yielded compound **2** which inhibited both proteases with higher potency than its linear congener—in the case of the West Nile virus by more than two orders of magnitude (Figures 4 and 5) [33].

Another effect that is often associated with increased conformational rigidity is elevated proteolytic stability [3, 13, 59]. Peptides with restricted conformational flexibility often have elongated half‐lives. Explanations for that may be found in the inadequate conformation of constrained peptides relative to the protease binding pocket, which tend to bind linear sections of a peptide and the higher energy barrier necessary to adopt a transition state during proteolytic digestion. Proteolytic stability studies of bismuth Bicycle molecules and their linear analogues (without bismuth) demonstrated that bismuth Bicycles were 6 to 19 times more resistant to proteolytic digestion (depending on the peptide's sequence and protease in question) (Figure 5) [33].

## 4 Cell‐Penetrating Bismuth Bicycle Molecules

In addition to improved biological activity and proteolytic stability, conformational constraint can also enhance the cellular uptake of cell‐penetrating peptides (CPPs) [60, 61, 62, 63, 64, 65, 66]. CPPs are short peptide sequences known to undergo cellular internalisation [60]. While mechanistic details remain the subject of ongoing research, a significant proportion of CPPs are believed to undergo receptor‐mediated endocytosis [60, 67, 68, 69, 70]. The majority of CPPs are either amphipathic or polycationic, such as the first reported examples penetratin [67] and Tat_49‐57_ [71, 72]. The resemblance of compound **2** to polycationic CPPs prompted follow‐up studies which examined its properties using cellular systems [50]. A fluorescently labelled analogue **3** (Figure 4) was incubated with three different cancer cell lines and in all cases showed time‐ and concentration‐dependent cellular uptake as demonstrated by live‐cell confocal microscopy and fluorescence‐activated cell sorting (FACS). Cellular uptake of **3** was observed at concentrations as low as 10 nM. Compound **3** also showed a 10‐fold higher (concentration‐dependent) cellular uptake than known CPPs such as Tat_49–57_ and R_8_ (Figure 5). Mechanistic studies suggest an energy‐dependent uptake that is sensitive to rottlerin inhibition, rottlerin being an inhibitor of micropinocytosis [50].

Despite the efficient uptake rates of investigated cell‐penetrating bismuth Bicycle molecules, a challenge in their clinical application, as is the case for many CPPs [60, 73], remains their entrapment within endo‐lysosomal compartments. A separate study investigated modifications to the CPP sequence that could promote the release of bismuth Bicycle molecules from these subcellular compartments into the cytosol [74]. These modifications included the introduction of non‐canonical amino acids and inversion of stereo centres which led to a two‐fold improvement in a functional assay in comparison to their benchmark sequence CPP12 [74, 75].

Measuring a peptide's cellular uptake using techniques such as confocal microscopy or FACS necessitates the addition of a fluorescent dye to a compound of interest [60]. This modification ultimately alters a peptide's properties in ways which are difficult to predict and limits the comparability between different dyes and data sets (e.g., compare size and physicochemical properties of **2** and **3** in Figure 4) [60]. Unlike bicyclic peptides formed though cysteine alkylation, bismuth Bicycles carry a heavy metal core. This offers unique opportunities to leverage orthogonal quantification methods such as inductively coupled plasma‐mass spectrometry (ICP‐MS) and X‐ray fluorescence microscopy (XFM).

ICP‐MS can accurately quantify metals from a wide range of matrices, including biological samples [76, 77], enabling quantification of labelled and unlabelled bismuth Bicycle molecules (Figure 4). A comparative screening of various bismuth Bicycle molecules with and without fluorescent labels showed that the label had generally a negative impact on cellular uptake of polycationic CPPs [50].

XFM on the other hand, does not only allow the quantification of metals from biological matrices but also their subcellular localisation [78, 79, 80, 81]. To this end, most modern applications of XFM rely on synchrotron radiation because of its tuneable and high spectral brightness source of X‐rays [78]. In XFM each element yields a characteristic fluorescence spectrum which enables the quantification and localisation of a variety of metals from complex matrices, simultaneously [78, 79, 80, 81]. In a follow‐up study XFM was leveraged alongside fluorescence microscopy to investigate cell‐penetrating bismuth Bicycle molecules including **3** [82]. Three sets of compounds were prepared based on different CPP motifs which were labelled with a coumarin, naphthalimide or rhodamine dye. To enable tracking of both bismuth and the dye using XFM, an analogue of each dye was synthesised that carried a bromine substitution. Bromine is like bismuth an XFM active element with negligible background signal in mammalian cells. Correlative multimodal optical and x‐ray fluorescence images demonstrate co‐localisation of bismuth, bromine and the fluorescent dye following cellular uptake and thus provide further insights into the behaviour of this compound class [82].

## 5 Phage‐Encoded Bismuth Bicycle Molecules

Genetically encoded combinatorial libraries provide access to a vast spectrum of structurally unique peptides, allowing for the discovery of binders against a range of biological targets. Two independent studies examined in a proof‐of‐concept the use of Bi^3+^ salts as reagents to modify phage display libraries [83, 84]. The studies used two different phage constructs in which the semi‐randomised peptide sequence was either an N‐terminal extension of the pIII [83] or pVIII [84] protein. The different library formats used followed the general formula (CX_n_CX_m_C) with X_n_ and X_m_ being 3–5 randomised amino acids for engineered pIII proteins (9 libraries in total) [83] or X_n_ and X_m_ being 4 randomised amino acids for engineered pVIII proteins (1 library) [84]. Both studies concluded that Bi^3+^ salts are compatible with the phage display technology and enable access to genetically encoded bismuth Bicycle molecules. Binders were enriched against two different model proteins, namely maltose binding protein [83] and streptavidin [84]. Regardless of the moderate affinities, both studies reported exemplars that bound their respective target with dissociation constants that were two orders of magnitude greater in the presence of bismuth than in its absence [83, 84]. Future work will have to show the ability of the technique to generate bismuth Bicycle molecules against clinically relevant targets.

## 6 Potential Applications

In comparison to related pnictogens like arsenic or antimony, bismuth is remarkably well tolerated, allowing for its use in medical applications. Examples include bismuth tripotassium dicitrate (Gastrodenol) or the over‐the‐counter drug bismuth subsalicylate (Pepto‐Bismol).

In response to the rise of antimicrobial resistance, metals have gained increasing attention [85]. Amongst the more promising metals is bismuth, which is also used to treat gastrointestinal infections of *Helicobacter pylori* [86, 87]. In addition to its clinical use, pre‐clinical studies have shown that bismuth can inhibit beta‐lactamases [88] and sensitise multi‐drug‐resistant bacterial strains, highlighting potential applications as metalloantibiotic [89].

The emergence of precision guided medicine spurred a renewal of interest in the targeted radiopharmaceutical space [90]. Radiopharmaceuticals that gained approval in the last decade include Lutathera (EMA 2017, FDA 2018) [91] and Pluvicto (EMA and FDA 2022) [92] which both utilise peptidic ligands to bind to their cognate receptors within tumours. The roadblock in this rapidly growing research area is the availability of peptidic ligands against protein targets beyond the small number for which natural ligands are known (e.g., PSMA or Somatostatin‐2 Receptor) [93, 94, 95]. Genetically encoded peptide libraries, such as phage‐display, overcome these limitations and have proven suitable in identifying novel ligands even against targets that were previously deemed *undruggable* [8]. Ligands emerging from these screenings enable the selective delivery of a given payload to disease tissue while ensuring rapid clearance from circulation, thereby improving both safety and efficacy of the therapy. Targeted alpha therapy (TAT) holds promise in the treatment of cancer. In TAT, a ligand directs alpha emitting radionuclides to cancer cells to deliver localised radiation [96, 97]. Bismuth‐213, a radioactive isotope with a half‐life of ~46 min, decays through two different pathways to bismuth‐209, emitting either way an alpha particle in the process [98, 99]. Thus, replacing ^209^Bi for alpha‐emitting ^213^Bi in bismuth Bicycle molecules may yield a new class of precision radiopharmaceuticals. The instant, selective and quantitative conversion of linear peptides into their corresponding bismuth Bicycle molecule is certainly a unique advantage that calls for further investigation.

## 7 Summary

In recent years, bismuth Bicycle molecules have emerged as a novel class of constraint peptides. Similarly to alkylating agents, bismuth(III) can link three thiols in peptides; however, unlike conventional reagents, bismuth Bicycle molecules form instantaneously at physiological pH, yield quantitative conversions and tolerate the reducing agent TCEP. This enables the facile synthesis of bismuth Bicycle molecules even from genetically‐encoded peptide libraries, such as phage display, which unlocks an unexplored chemical space of compounds with unique properties for a range of applications including infectious diseases and cancer (Figure 5).

## Funding

Research supported by UK Research and Innovation (10090595).

## Conflicts of Interest

R.J.L.H., L.C., I.R., M.F., M.J.S. are employees and shareholders at Bicycle Therapeutics. S.V., A.S., A.T., D.R.S. declare no competing financial interest.

## Supporting information


**Data S1:** Supporting information.

## Data Availability

Primary research results supporting this article have been included in the article and its supplementary information.

## References

[1] W. Wang , E. Q. Wang , and J. P. Balthasar , “Monoclonal Antibody Pharmacokinetics and Pharmacodynamics,” Clinical Pharmacology and Therapeutics 84, no. 5 (2008): 548–558, 10.1038/clpt.2008.170. 18784655

[2] D. A. Smith , K. Beaumont , T. S. Maurer , and L. Di , “Relevance of Half‐Life in Drug Design,” Journal of Medicinal Chemistry 61, no. 10 (2018): 4273–4282, 10.1021/acs.jmedchem.7b00969. 29112446

[3] C. A. Rhodes and D. Pei , “Bicyclic Peptides as Next‐Generation Therapeutics,” Chemistry 23, no. 52 (2017): 12690–12703, 10.1002/chem.201702117. 28590540 PMC5603421

[4] M. Muttenthaler , G. F. King , D. J. Adams , and P. F. Alewood , “Trends in Peptide Drug Discovery,” Nature Reviews. Drug Discovery 20, no. 4 (2021): 309–325, 10.1038/s41573-020-00135-8. 33536635

[5] L. Wang , N. Wang , W. Zhang , et al., “Therapeutic Peptides: Current Applications and Future Directions,” Signal Transduction and Targeted Therapy 7, no. 1 (2022): 48, 10.1038/s41392-022-00904-4. 35165272 PMC8844085

[6] D. Feng , L. Liu , Y. Shi , et al., “Current Development of Bicyclic Peptides,” Chinese Chemical Letters 34, no. 6 (2023): 108026, 10.1016/j.cclet.2022.108026.

[7] K. Colas , D. Bindl , and H. Suga , “Selection of Nucleotide‐Encoded Mass Libraries of Macrocyclic Peptides for Inaccessible Drug Targets,” Chemical Reviews 124, no. 21 (2024): 12213–12241, 10.1021/acs.chemrev.4c00422. 39451037 PMC11565579

[8] M. El Fakiri , A. R. Regupathy , L. Uhlmann , et al., “Development and Preclinical Characterization of a Novel Radiotheranostic EphA2‐Targeting Bicyclic Peptide,” Theranostics 14, no. 12 (2024): 4701–4712, 10.7150/thno.96641. 39239524 PMC11373624

[9] M. M. Schmidt and K. D. Wittrup , “A Modeling Analysis of the Effects of Molecular Size and Binding Affinity on Tumor Targeting,” Molecular Cancer Therapeutics 8, no. 10 (2009): 2861–2871, 10.1158/1535-7163.MCT-09-0195. 19825804 PMC4078872

[10] G. E. Mudd , A. Brown , L. Chen , et al., “Identification and Optimization of EphA2‐Selective Bicycles for the Delivery of Cytotoxic Payloads,” Journal of Medicinal Chemistry 63, no. 8 (2020): 4107–4116, 10.1021/acs.jmedchem.9b02129. 32202781

[11] G. E. Mudd , H. Scott , L. Chen , et al., “Discovery of BT8009: a Nectin‐4 Targeting Bicycle Toxin Conjugate for the Treatment of Cancer,” Journal of Medicinal Chemistry 65, no. 21 (2022): 14337–14347, 10.1021/acs.jmedchem.2c00065. 36204777 PMC9661471

[12] S. Ullrich and C. Nitsche , “Bicyclic Peptides: Paving the Road for Therapeutics of the Future,” Peptide Science 116, no. 2 (2024): e24326, 10.1002/pep2.24326.

[13] T. A. Hill , N. E. Shepherd , F. Diness , and D. P. Fairlie , “Constraining Cyclic Peptides to Mimic Protein Structure Motifs,” Angewandte Chemie, International Edition 53, no. 48 (2014): 13020–13041, 10.1002/anie.201401058. 25287434

[14] V. J. Thombare and C. A. Hutton , “Bridged Bicyclic Peptides: Structure and Function,” Peptide Science 110, no. 3 (2018): e24057, 10.1002/pep2.24057.

[15] F. J. Chen , N. Pinnette , and J. Gao , “Strategies for the Construction of Multicyclic Phage Display Libraries,” Chembiochem 25, no. 9 (2024): e202400072, 10.1002/cbic.202400072. 38466139 PMC11437370

[16] C. Heinis , T. Rutherford , S. Freund , and G. Winter , “Phage‐Encoded Combinatorial Chemical Libraries Based on Bicyclic Peptides,” Nature Chemical Biology 5, no. 7 (2009): 502–507, 10.1038/nchembio.184. 19483697

[17] P. Timmerman , J. Beld , W. C. Puijk , and R. H. Meloen , “Rapid and Quantitative Cyclisation of Multiple Peptide Loops Onto Synthetic Scaffolds for Structural Mimicry of Protein Surfaces,” Chembiochem 6, no. 5 (2005): 821–824, 10.1002/cbic.200400374. 15812852

[18] C. Heinis and G. Winter , “Encoded libraries of chemically modified peptides,” Current Opinion in Chemical Biology 26 (2015): 89–98, 10.1016/j.cbpa.2015.02.008. 25768886

[19] X. Ji , A. L. Nielsen , and C. Heinis , “Cyclic Peptides for Drug Development,” Angewandte Chemie, International Edition 63, no. 3 (2024): e202308251, 10.1002/anie.202308251. 37870189

[20] M. Rigby , G. Bennett , L. Chen , et al., “BT8009; A Nectin‐4 Targeting Bicycle Toxin Conjugate for Treatment of Solid Tumors,” Molecular Cancer Therapeutics 21, no. 12 (2022): 1747–1756, 10.1158/1535-7163.MCT-21-0875. 36112771 PMC9940631

[21] C. Baldini , V. Goldschmidt , I. Brana , et al., “BT8009‐100: A Phase I/II Study of Novel Bicyclic Peptide and MMAE Conjugate BT8009 in Patients (pts) With Advanced Malignancies Associated With Nectin‐4 Expression, Including Urothelial Cancer (UC),” Journal of Clinical Oncology 41, no. 6_suppl (2023): 498–498, 10.1200/JCO.2023.41.6_suppl.498.

[22] K. P. Papadopoulos , A. Dowlati , A. Dickson , et al., “A Combined Phase I/II Study of a Novel Bicycle Tumor‐Targeted Immune Cell Agonist BT7480 in Patients With Nectin‐4 Associated Advanced Malignancies,” Journal of Clinical Oncology 40, no. 16_suppl (2022): TPS2689, 10.1200/JCO.2022.40.16_suppl.TPS2689.

[23] J. C. Bendell , J. S.‐Z. Wang , B. Bashir , et al., “BT5528‐100 Phase I/II Study of the Safety, Pharmacokinetics, and Preliminary Clinical Activity of BT5528 in Patients With Advanced Malignancies Associated With EphA2 Expression,” Journal of Clinical Oncology 38, no. 15_suppl (2020): TPS3655, 10.1200/JCO.2020.38.15_suppl.TPS3655.

[24] B. Bashir , J. S. Wang , G. Falchook , et al., “Results From First‐In‐Human Phase I Dose‐Escalation Study of a Novel Bicycle Toxin Conjugate Targeting EphA2 (BT5528) in Patients With Advanced Solid Tumors,” Journal of Clinical Oncology 42, no. 29 (2024): 3443–3452, 10.1200/jco.23.01107. 39231383

[25] G. Bennett , A. Brown , G. Mudd , et al., “MMAE Delivery Using the Bicycle Toxin Conjugate BT5528,” Molecular Cancer Therapeutics 19, no. 7 (2020): 1385–1394, 10.1158/1535-7163.MCT-19-1092. 32398269

[26] S. Chen , D. Bertoldo , A. Angelini , F. Pojer , and C. Heinis , “Peptide Ligands Stabilised by Small Molecules,” Angewandte Chemie, International Edition 53, no. 6 (2014): 1602–1606, 10.1002/anie.201309459. 24453110

[27] S. Chen , J. Morales‐Sanfrutos , A. Angelini , B. Cutting , and C. Heinis , “Structurally Diverse Cyclisation Linkers Impose Different Backbone Conformations in Bicyclic Peptides,” Chembiochem 13, no. 7 (2012): 1032–1038, 10.1002/cbic.201200049. 22492661

[28] K. Deyle , X. D. Kong , and C. Heinis , “Phage Selection of Cyclic Peptides for Application in Research and Drug Development,” Accounts of Chemical Research 50, no. 8 (2017): 1866–1874, 10.1021/acs.accounts.7b00184. 28719188

[29] K. U. Gaynor , M. Vaysburd , M. A. J. Harman , et al., “Multivalent Bicyclic Peptides Are an Effective Antiviral Modality That Can Potently Inhibit SARS‐CoV‐2,” Nature Communications 14, no. 1 (2023): 3583, 10.1038/s41467-023-39158-1. PMC10275983 37328472

[30] G. E. Mudd , S. J. Stanway , D. R. Witty , et al., “Gold‐Mediated Multiple Cysteine Arylation for the Construction of Highly Constrained Bicycle Peptides,” Bioconjugate Chemistry 33, no. 8 (2022): 1441–1445, 10.1021/acs.bioconjchem.2c00288. 35894801

[31] J. T. Hampton and W. R. Liu , “Diversification of Phage‐Displayed Peptide Libraries With Noncanonical Amino Acid Mutagenesis and Chemical Modification,” Chemical Reviews 124, no. 9 (2024): 6051–6077, 10.1021/acs.chemrev.4c00004. 38686960 PMC11082904

[32] P. Diderich and C. Heinis , “Directed Evolution of Bicyclic Peptides for Therapeutic Application,” Chimia 67, no. 12‐13 (2013): 910–915, 10.2533/chimia.2013.910. 24594337

[33] S. Voss , J. Rademann , and C. Nitsche , “Peptide‐Bismuth Bicycles: In‐Situ Access to Stable Constrained Peptides With Superior Bioactivity,” Angewandte Chemie International Edition 61, no. 4 (2022): e202113857, 10.1002/anie.202113857. 34825756

[34] H. G. de Carvalho and M. de Araújo Penna , “Alpha‐Activity of ^209^Bi,” Lettere al Nuovo Cimento 3, no. 18 (1972): 720–722, 10.1007/BF02824346.

[35] P. de Marcillac , N. Coron , G. Dambier , J. Leblanc , and J. P. Moalic , “Experimental Detection of Alpha‐Particles From the Radioactive Decay of Natural Bismuth,” Nature 422, no. 6934 (2003): 876–878, 10.1038/nature01541. 12712201

[36] N. Yang and H. Sun , “Biocoordination Chemistry of Bismuth: Recent Advances,” Coordination Chemistry Reviews 251, no. 17 (2007): 2354–2366, 10.1016/j.ccr.2007.03.003.

[37] H. Sun , H. Li , and P. J. Sadler , “The Biological and Medicinal Chemistry of Bismuth,” Chemische Berichte 130, no. 6 (1997): 669–681, 10.1002/cber.19971300602.

[38] R. Ge and H. Sun , “Bioinorganic Chemistry of Bismuth and Antimony: Target Sites of Metallodrugs,” Accounts of Chemical Research 40, no. 4 (2007): 267–274, 10.1021/ar600001b. 17330963

[39] Y. J. Wang and L. Xu , “pH‐Dependent Displacement of [Bi(citrate)](‐) With Cysteine: Synthesis, Spectroscopic and X‐Ray Crystallographic Characterization of Bi(cysteine)_3_ ,” Journal of Inorganic Biochemistry 102, no. 4 (2008): 988–991, 10.1016/j.jinorgbio.2008.01.004. 18262279

[40] G. G. Briand , N. Burford , M. D. Eelman , et al., “Identification, Isolation, and Characterization of Cysteinate and Thiolactate Complexes of Bismuth,” Inorganic Chemistry 43, no. 20 (2004): 6495–6500, 10.1021/ic049594n. 15446902

[41] P. J. Sadler , H. Sun , and H. Li , “Bismuth(III) Complexes of the Tripeptide Glutathione (γ‐L‐Glu–L‐Cys–Gly),” Chemistry ‐ A European Journal 2, no. 6 (1996): 701–708, 10.1002/chem.19960020615.

[42] S. Potocki , M. Rowinska‐Zyrek , D. Valensin , et al., “Metal Binding Ability of Cysteine‐Rich Peptide Domain of ZIP13 Zn2+ Ions Transporter,” Inorganic Chemistry 50, no. 13 (2011): 6135–6145, 10.1021/ic200270p. 21630642

[43] J. J. R. Frausto da Silva , “The Chelate Effect Redefined,” Journal of Chemical Education 60, no. 5 (1983): 390, 10.1021/ed060p390.

[44] M. Matzapetakis , D. Ghosh , T. C. Weng , J. E. Penner‐Hahn , and V. L. Pecoraro , “Peptidic Models for the Binding of Pb(II), Bi(III) and Cd(II) to Mononuclear Thiolate Binding Sites,” Journal of Biological Inorganic Chemistry 11, no. 7 (2006): 876–890, 10.1007/s00775-006-0140-7. 16855818

[45] S. Cun , H. Li , R. Ge , M. C. M. Lin , and H. Sun , “A Histidine‐Rich and Cysteine‐Rich Metal‐Binding Domain at the C‐Terminus of Heat Shock Protein a From *Helicobacter pylori* : Implications for Nickel Homeostatis and Bismuth Susceptibility,” Journal of Biological Chemistry 283, no. 22 (2008): 15142–15151, 10.1074/jbc.M800591200. 18364351 PMC3258894

[46] M. Rowinska‐Zyrek , D. Valensin , L. Szyrwiel , Z. Grzonka , and H. Kozlowski , “Specific Interactions of Bi(III) With the Cys‐Xaa‐Cys Unit of a Peptide Sequence,” Dalton Transactions 42 (2009): 9131–9140, 10.1039/b913430a. 20449188

[47] M. Rowinska‐Zyrek , D. Witkowska , D. Valensin , W. Kamysz , and H. Kozlowski , “The C Terminus of HspA‐‐A Potential Target for Native Ni(II) and Bi(III) Anti‐Ulcer Drugs,” Dalton Transactions 39, no. 25 (2010): 5814–5826, 10.1039/c0dt00013b. 20502777

[48] C. Nitsche , M. C. Mahawaththa , W. Becker , T. Huber , and G. Otting , “Site‐Selective Tagging of Proteins by Pnictogen‐Mediated Self‐Assembly,” Chemical Communications 53, no. 79 (2017): 10894–10897, 10.1039/c7cc06155b. 28890978

[49] B. Cordero , V. Gomez , A. E. Platero‐Prats , et al., “Covalent Radii Revisited,” Dalton Transactions 21 (2008): 2832–2838, 10.1039/b801115j. 18478144

[50] S. Voss , L. D. Adair , K. Achazi , et al., “Cell‐Penetrating Peptide‐Bismuth Bicycles,” Angewandte Chemie International Edition 63, no. 10 (2024): e202318615, 10.1002/anie.202318615. 38126926

[51] L. J. Davies , P. Ghosh , S. Siryer , S. Ullrich , and C. Nitsche , “Peptide‐Bismuth Tricycles: Maximizing Stability by Constraint,” Chemistry 31, no. 8 (2025): e202500064, 10.1002/chem.202500064. 39803821

[52] P. Ghosh , M. Shang , K. Trajkovic , et al., “Bismuth‐Selenopeptides Combine Potent Bioactivity With Exceptional Kinetic Inertness,” Angewandte Chemie International Edition 64, no. 46 (2025): e202517700, 10.1002/anie.202517700. 41024505 PMC12603966

[53] J. Ramler and C. Lichtenberg , “Molecular Bismuth Cations: Assessment of Soft Lewis Acidity,” Chemistry ‐ A European Journal 26, no. 45 (2020): 10250–10258, 10.1002/chem.202001674. 32428329 PMC7818483

[54] R. G. Pearson , “Hard and Soft Acids and Bases, HSAB, Part 1: Fundamental Principles,” Journal of Chemical Education 45, no. 9 (1968): 581, 10.1021/ed045p581.

[55] R. G. Pearson , “Hard and Soft Acids and Bases, HSAB, Part II: Underlying Theories,” Journal of Chemical Education 45, no. 10 (1968): 643, 10.1021/ed045p643.

[56] H. Kessler , “Conformation and Biological Activity of Cyclic Peptides,” Angewandte Chemie, International Edition 21, no. 7 (1982): 512–523, 10.1002/anie.198205121.

[57] S. Voss and C. Nitsche , “Inhibitors of the Zika Virus Protease NS2B‐NS3,” Bioorganic & Medicinal Chemistry Letters 30, no. 5 (2020): 126965, 10.1016/j.bmcl.2020.126965. 31980339

[58] S. Voss and C. Nitsche , “Targeting the Protease of West Nile Virus,” RSC Medicinal Chemistry 12, no. 8 (2021): 1262–1272, 10.1039/d1md00080b. 34458734 PMC8372202

[59] B. Khatri , V. R. Nuthakki , and J. Chatterjee , “Strategies to Enhance Metabolic Stabilities,” Methods in Molecular Biology 2001 (2019): 17–40, 10.1007/978-1-4939-9504-2_2. 31134565

[60] P. G. Dougherty , A. Sahni , and D. Pei , “Understanding cell penetration of cyclic peptides,” Chemical Reviews 119, no. 17 (2019): 10241–10287, 10.1021/acs.chemrev.9b00008. 31083977 PMC6739158

[61] N. Nischan , H. D. Herce , F. Natale , et al., “Covalent Attachment of Cyclic TAT Peptides to GFP Results in Protein Delivery Into Live Cells With Immediate Bioavailability,” Angewandte Chemie, International Edition 54, no. 6 (2015): 1950–1953, 10.1002/anie.201410006. 25521313

[62] A. F. L. Schneider , M. Kithil , M. C. Cardoso , M. Lehmann , and C. P. R. Hackenberger , “Cellular Uptake of Large Biomolecules Enabled by Cell‐Surface‐Reactive Cell‐Penetrating Peptide Additives,” Nature Chemistry 13, no. 6 (2021): 530–539, 10.1038/s41557-021-00661-x. 33859390

[63] O. Tietz , F. Cortezon‐Tamarit , R. Chalk , S. Able , and K. A. Vallis , “Tricyclic Cell‐Penetrating Peptides for Efficient Delivery of Functional Antibodies Into Cancer Cells,” Nature Chemistry 14, no. 3 (2022): 284–293, 10.1038/s41557-021-00866-0. PMC7617065 35145246

[64] C. A. Rhodes , P. G. Dougherty , J. K. Cooper , et al., “Cell‐Permeable Bicyclic Peptidyl Inhibitors Against NEMO‐IkappaB Kinase Interaction Directly From a Combinatorial Library,” Journal of the American Chemical Society 140, no. 38 (2018): 12102–12110, 10.1021/jacs.8b06738. 30176143 PMC6231237

[65] W. Lian , B. Jiang , Z. Qian , and D. Pei , “Cell‐Permeable Bicyclic Peptide Inhibitors Against Intracellular Proteins,” Journal of the American Chemical Society 136, no. 28 (2014): 9830–9833, 10.1021/ja503710n. 24972263 PMC4227718

[66] J. M. Wolfe , C. M. Fadzen , R. L. Holden , M. Yao , G. J. Hanson , and B. L. Pentelute , “Perfluoroaryl Bicyclic Cell‐Penetrating Peptides for Delivery of Antisense Oligonucleotides,” Angewandte Chemie, International Edition 57, no. 17 (2018): 4756–4759, 10.1002/anie.201801167. 29479836 PMC6248909

[67] G. Guidotti , L. Brambilla , and D. Rossi , “Cell‐Penetrating Peptides: From Basic Research to Clinics,” Trends in Pharmacological Sciences 38, no. 4 (2017): 406–424, 10.1016/j.tips.2017.01.003. 28209404

[68] H. Derakhshankhah and S. Jafari , “Cell penetrating peptides: a concise review with emphasis on biomedical applications,” Biomedicine & Pharmacotherapy 108 (2018): 1090–1096, 10.1016/j.biopha.2018.09.097. 30372809

[69] I. Ruseska and A. Zimmer , “Internalisation Mechanisms of Cell‐Penetrating Peptides,” Beilstein Journal of Nanotechnology 11 (2020): 101–123, 10.3762/bjnano.11.10. 31976201 PMC6964662

[70] A. Gori , G. Lodigiani , S. G. Colombarolli , G. Bergamaschi , and A. Vitali , “Cell Penetrating Peptides: Classification, Mechanisms, Methods of Study and Applications,” ChemMedChem 18, no. 17 (2023): e202300236, 10.1002/cmdc.202300236. 37389978

[71] M. Green and P. M. Loewenstein , “Autonomous Functional Domains of Chemically Synthesized Human Immunodeficiency Virus Tat Trans‐Activator Protein,” Cell 55, no. 6 (1988): 1179–1188, 10.1016/0092-8674(88)90262-0. 2849509

[72] A. Joliot , C. Pernelle , H. Deagostini‐Bazin , and A. Prochiantz , “Antennapedia Homeobox Peptide Regulates Neural Morphogenesis,” Proceedings of the National Academy of Sciences 88, no. 5 (1991): 1864–1868, 10.1073/pnas.88.5.1864. PMC51126 1672046

[73] D. Pei and M. Buyanova , “Overcoming Endosomal Entrapment in Drug Delivery,” Bioconjugate Chemistry 30, no. 2 (2019): 273–283, 10.1021/acs.bioconjchem.8b00778. 30525488 PMC6501178

[74] J. L. Ritchey , L. Filippi , D. Ballard , and D. Pei , “Bismuth‐Cyclized Cell‐Penetrating Peptides,” Molecular Pharmaceutics 21, no. 10 (2024): 5255–5260, 10.1021/acs.molpharmaceut.4c00688. 39223839 PMC11610496

[75] M. Buyanova , A. Sahni , R. Yang , A. Sarkar , H. Salim , and D. Pei , “Discovery of a Cyclic Cell‐Penetrating Peptide With Improved Endosomal Escape and Cytosolic Delivery Efficiency,” Molecular Pharmaceutics 19, no. 5 (2022): 1378–1388, 10.1021/acs.molpharmaceut.1c00924. 35405068 PMC9175492

[76] A. A. Ammann , “Inductively Coupled Plasma‐Mass Spectrometry (ICP‐MS): A Versatile Tool,” Journal of Mass Spectrometry 42, no. 4 (2007): 419–427, 10.1002/jms.1206. 17385793

[77] U. Gießmann and U. Greb , “High Resolution ICP‐MS—A New Concept for Elemental Mass Spectrometry,” Fresenius Journal of Analytical Chemistry 350, no. 4 (1994): 186–193, 10.1007/BF00322469.

[78] M. J. Pushie , I. J. Pickering , M. Korbas , M. J. Hackett , and G. N. George , “Elemental and Chemically Specific X‐Ray Fluorescence Imaging of Biological Systems,” Chemical Reviews 114, no. 17 (2014): 8499–8541, 10.1021/cr4007297. 25102317 PMC4160287

[79] M. J. Pushie , N. J. Sylvain , H. Hou , M. J. Hackett , M. E. Kelly , and S. M. Webb , “X‐Ray Fluorescence Microscopy Methods for Biological Tissues,” Metallomics 14, no. 6 (2022): mfac032, 10.1093/mtomcs/mfac032. 35512669 PMC9226457

[80] C. J. Fahrni , “Biological Applications of X‐Ray Fluorescence Microscopy: Exploring the Subcellular Topography and Speciation of Transition Metals,” Current Opinion in Chemical Biology 11, no. 2 (2007): 121–127, 10.1016/j.cbpa.2007.02.039. 17353139

[81] S. Vogt and A. Lanzirotti , “Trends in X‐Ray Fluorescence Microscopy,” Synchrotron Radiation News 26, no. 2 (2013): 32–38, 10.1080/08940886.2013.771072.

[82] S. Voss , C. Kidman , L. D. Adair , et al., “Triple Threat Bismuth Peptide Imaging in Cells,” preprint, ChemRxiv August 27, 2025, 10.26434/chemrxiv-2025-w9pdn. PMC12959375 41789354

[83] R. N. He , M. J. Zhang , B. Dai , and X. D. Kong , “Selection of Peptide‐Bismuth Bicycles Using Phage Display,” ACS Chemical Biology 19, no. 5 (2024): 1040–1044, 10.1021/acschembio.4c00099. 38620022

[84] S. Ullrich , U. Somathilake , M. Shang , and C. Nitsche , “Phage‐Encoded Bismuth Bicycles Enable Instant Access to Targeted Bioactive Peptides,” Communications Chemistry 7, no. 1 (2024): 143, 10.1038/s42004-024-01232-0. 38937646 PMC11211329

[85] A. Frei , A. D. Verderosa , A. G. Elliott , J. Zuegg , and M. A. T. Blaskovich , “Metals to Combat Antimicrobial Resistance,” Nature Reviews Chemistry 7, no. 3 (2023): 202–224, 10.1038/s41570-023-00463-4. 37117903 PMC9907218

[86] H. Alkim , A. R. Koksal , S. Boga , I. Sen , and C. Alkim , “Role of Bismuth in the Eradication of *Helicobacter pylori* ,” American Journal of Therapeutics 24, no. 6 (2017): e751–e757, 10.1097/MJT.0000000000000389. 26808355

[87] M. P. Dore , H. Lu , and D. Y. Graham , “Role of Bismuth in Improving *Helicobacter pylori* Eradication With Triple Therapy,” Gut 65, no. 5 (2016): 870–878, 10.1136/gutjnl-2015-311019. 26848181

[88] R. Wang , T. P. Lai , P. Gao , et al., “Bismuth Antimicrobial Drugs Serve as Broad‐Spectrum Metallo‐Beta‐Lactamase Inhibitors,” Nature Communications 9, no. 1 (2018): 439, 10.1038/s41467-018-02828-6. PMC5789847 29382822

[89] Y. Xia , X. Wei , P. Gao , et al., “Bismuth‐Based Drugs Sensitise *Pseudomonas aeruginosa* to Multiple Antibiotics by Disrupting Iron Homeostasis,” Nature Microbiology 9, no. 10 (2024): 2600–2613, 10.1038/s41564-024-01807-6. 39294461

[90] S. Zhang , X. Wang , X. Gao , et al., “Radiopharmaceuticals and Their Applications in Medicine,” Signal Transduction and Targeted Therapy 10, no. 1 (2025): 1, 10.1038/s41392-024-02041-6. 39747850 PMC11697352

[91] U. Hennrich and K. Kopka , “Lutathera: The First FDA‐ and EMA‐Approved Radiopharmaceutical for Peptide Receptor Radionuclide Therapy,” Pharmaceuticals 12, no. 3 (2019): 114, 10.3390/ph12030114. 31362406 PMC6789871

[92] U. Hennrich and M. Eder , “Lu‐PSMA‐617 Pluvicto: the First FDA‐Approved Radiotherapeutical for Treatment of Prostate Cancer,” Pharmaceuticals. 15, no. 10 (2022): 1292, 10.3390/ph15101292. 36297404 PMC9608311

[93] L. Bodei , K. Herrmann , H. Schoder , A. M. Scott , and J. S. Lewis , “Radiotheranostics in Oncology: Current Challenges and Emerging Opportunities,” Nature Reviews. Clinical Oncology 19, no. 8 (2022): 534–550, 10.1038/s41571-022-00652-y. PMC10585450 35725926

[94] M. Shabsigh and L. A. Solomon , “Peptide PET Imaging: A Review of Cecent Developments and a Look at the Future of Radiometal‐Labeled Peptides in Medicine,” Chemical & Biomedical Imaging 2, no. 9 (2024): 615–630, 10.1021/cbmi.4c00030. 39474267 PMC11503725

[95] L. Aloj , R. Mansi , S. De Luca , A. Accardo , D. Tesauro , and G. Morelli , “Radiolabeled Peptides and Their Expanding Role in Clinical Imaging and Targeted Cancer Therapy,” Journal of Peptide Science 30, no. 10 (2024): e3607, 10.1002/psc.3607. 38710638

[96] Y. S. Kim and M. W. Brechbiel , “An Overview of Targeted Alpha Therapy,” Tumour Biology 33, no. 3 (2012): 573–590, 10.1007/s13277-011-0286-y. 22143940 PMC7450491

[97] F. D. C. Guerra Liberal , J. M. O'Sullivan , S. J. McMahon , and K. M. Prise , “Targeted Alpha Therapy: Current Clinical Applications,” Cancer Biotherapy & Radiopharmaceuticals 35, no. 6 (2020): 404–417, 10.1089/cbr.2020.3576. 32552031

[98] S. Ahenkorah , I. Cassells , C. M. Deroose , et al., “Bismuth‐213 for Targeted Radionuclide Therapy: From Atom to Bedside,” Pharmaceutics 13, no. 5 (2021): 599, 10.3390/pharmaceutics13050599. 33919391 PMC8143329

[99] F. Bruchertseifer , A. Kellerbauer , R. Malmbeck , and A. Morgenstern , “Targeted Alpha Therapy With Bismuth‐213 and Actinium‐225: Meeting Future Demand,” Journal of Labelled Compounds and Radiopharmaceuticals 62, no. 11 (2019): 794–802, 10.1002/jlcr.3792. 31369165

