# Supplementary material for: Bismuth Bicycles
Source: J Pept Sci. 2026 Feb 3;32(3):e70071. doi: 10.1002/psc.70071 (PMC12868410; doi:10.1002/psc.70071)
Supplement: Supplementary file 1 — Data S1: Supporting information. [file PSC-32-e70071-s001.docx]

**Bismuth Bicycles**

**Supporting information**

Saan Voss,* Amin Sagar, Arnaud Tiberghien, Richard J. L. Hughes, Liuhong Chen, Inmaculada Rioja, Mark Frigerio, Michael J. Skynner, David R. Spring

*Corresponding author: srv26@cam.ac.uk

**Conformational sampling**

**Methods**

We generated the conformational ensembles for the Bi^3+^, TBMB, TATA, TBAB and TATB bound to three N-acetyl cysteine methyl ester using the iterative meta-dynamics with genetic crossing (iMTD-GC) method as implemented in the CREST software package.^1^ All the calculations were performed using the GFN-FF method with the length of the simulations determined automatically based on the flexibility of the input molecule. All structures found within a range of 6 kcal/mol of the lowest energy conformer were saved and analysed for distances between the C-alpha atoms of the cysteines and Bismuth atom or the centroid of the central rings of the organic scaffolds (TBMB, TATA, TBAB and TATB) using MDAnalysis.^2, 3^ The distance distributions were plotted with seaborn library as density by normalizing the plots so that the total area of the histograms equals 1 in all cases.

**Results**

We explored the sizes and flexibility of various scaffolds by sampling their conformations using iterative meta-dynamics with genetic crossing (iMTD-GC) simulations using the CREST software package. The distances between the C-alpha atoms and the Bismuth atom or the centroid of the central rings in the other scaffolds is plotted in Figure 3. Further details can be found in the main article.

**References**

(1) Pracht, P.; Bohle, F.; Grimme, S.; Automated exploration of the low-energy chemical space with fast quantum chemical methods. *Phys. Chem. Chem. Phys.,* **2020**, *22* (14), 7169-7192, DOI: 10.1039/c9cp06869d.

(2) Michaud-Agrawal, N.; Denning, E. J.; Woolf, T. B.; Beckstein, O.; MDAnalysis: A toolkit for the analysis of molecular dynamics simulations. *J. Comput. Chem.,* **2011**, *32* (10), 2319-2327, DOI: 10.1002/jcc.21787.

(3) Gowers, R.; Linke, M.; Barnoud, J.; Reddy, T.; Melo, M.; Seyler, S. L.; Domański, J.; Dotson, D.; Buchoux, S.; Kenney, I.; Beckstein, O. MDAnalysis: A python package for the rapid analysis of molecular dynamics simulations. In Proceeding of the 15th python in science conference (SCIPY), 2016.
